# Lymphatic filariasis elimination in the Dominican Republic: History, progress, and remaining steps

**DOI:** 10.1371/journal.pntd.0009590

**Published:** 2021-08-10

**Authors:** Manuel Gonzales, Gregory S. Noland, Eileen F. Mariano, Stephen Blount

**Affiliations:** 1 Centro de Prevención y Control de Enfermedades Transmitidas por Vectores y Zoonosis, Ministerio de Salud Pública, Santo Domingo, The Dominican Republic; 2 The Carter Center, Atlanta, Georgia, United States of America; Erasmus MC, NETHERLANDS

## Abstract

Lymphatic filariasis (LF) is a mosquito-transmitted parasitic disease that is a leading cause of disability globally. The island of Hispaniola, which the Dominican Republic shares with Haiti, accounts for approximately 90% of LF cases in the Americas region. In 1998, the Dominican Ministry of Public Health created the Program to Eliminate Lymphatic Filariasis (PELF) with the goal of eliminating LF transmission by 2020. Baseline mapping revealed 19 (12% of total) endemic municipalities clustered into three geographic foci (Southwest, La Ciénaga and East), with a total at-risk population of 262,395 people. Beginning in 2002, PELF sequentially implemented mass drug administration (MDA) in these foci using albendazole and diethylcarbamazine (DEC). In total, 1,174,050 treatments were given over three to five annual rounds of house-to-house MDA per focus with a median coverage of 81.7% (range 67.4%–92.2%). By 2018, LF antigen prevalence was less than 2% in all foci, thus meeting criteria to stop MDA and begin post-treatment surveillance (PTS). This success has been achieved against a shifting landscape of limited domestic funding, competing domestic public health priorities, and sporadic external donor support. Remaining steps include the need to scale-up morbidity management and disability prevention services for LF and to continue PTS until LF transmission is interrupted across Hispaniola.

## Author summary

Lymphatic filariasis (LF) is a neglected tropical disease caused by thin worms transmitted to humans by the bites of mosquitoes. LF is a leading cause of disability globally, resulting from the long-term physical (swelling of legs and genitals) and psycho-social impacts on affected individuals. The Dominican Republic is one of four LF-endemic countries in the Americas. This report describes the efforts to eliminate the disease in the country, including a review of disease mapping and implementation of mass drug administration (MDA)—the provision of safe and effective medicine to at-risk communities—to interrupt parasite transmission. Particular emphasis is given to program planning and community mobilization for MDA due to the divergent environments of endemic foci, which ranged from rural agricultural regions to densely populated urban neighborhoods. Impact evaluation data for each focus demonstrates interruption of LF transmission and that criteria to stop MDA have been met. Finally, plans for post-treatment surveillance and the scale-up of clinical care services for those who remain affected by chronic LF are presented—required elements for eliminating LF as a public health problem.

## Introduction

Lymphatic filariasis (LF) is a mosquito-transmitted neglected tropical disease with an estimated 858 million people at risk in 72 endemic countries [1]. LF is caused by infection with one of three species of filarial nematodes that induce lymphatic dysfunction resulting in lymphedema, elephantiasis, and male genital swelling (hydrocele) [2]. These conditions lead to reduced mobility, impairment of daily activities, and social isolation for affected individuals [3,4] claiming at least 1.3 million disability-adjusted life years [5]. In 1993, the International Task Force for Disease Eradication declared LF one of six eradicable diseases [6]. The World Health Assembly called for the elimination of LF as a public health problem in 1997 [7], followed by the launching in 2000 of the Global Programme to Eliminate Lymphatic Filariasis (GPELF) by the World Health Organization (WHO). The global strategy consists of mass drug administration (MDA) to interrupt parasite transmission and supportive care to alleviate disability for those already affected by LF. The drugs used for MDA—albendazole (donated by GlaxoSmithKline) co-administered with either diethylcarbamazine (DEC, donated by Eisai) or ivermectin (Mectizan, donated by Merck & Co.)—reduce the number of viable infectious stage microfilariae (MF) found in circulation of the human host, thereby reducing risk of transmission to mosquitoes. Annual MDA for 4–6 years at effective coverage (≥ 65%) in at-risk populations is predicted to reduce infection prevalence to levels below which transmission is no longer sustainable [8].

The Dominican Republic (population 10.2 million) is one of only four LF-endemic countries in the Americas, and the island of Hispaniola, which the Dominican Republic shares with Haiti, accounts for approximately 90% of cases in the region [9]. In Hispaniola, LF is caused by *Wuchereria bancrofti* with *Culex quinquefasciatus* the principal vector [10]. In 1998, the Dominican Ministry of Public Health established the Programa de Eliminación de la Filariasis Linfática (PELF) to accomplish the following goals: 1) to eliminate LF transmission in the country by 2020; and 2) to ensure availability of morbidity management and disability prevention (MMDP) services for those who suffer from LF. Publications have highlighted the progress of LF elimination in neighboring Haiti [11,12]. This review summarizes the progress toward achieving these goals in the Dominican Republic.

## Materials and methods

### Ethics statement

Surveys or other assessments conducted by PELF and reported in this manuscript were conducted as non-research public health activities of the Dominican Ministry of Health. For all surveys, written informed assent or consent was obtained for any survey participant. The 2009 and 2012 post-treatment surveillance surveys in the Southwest focus were approved by the Dominican Consejo Nacional de Bioética en Salud [13] and the 2014 transmission assessment survey in La Ciénaga was deemed non-human subjects research by Emory Institutional Review Board [14].

### Data sources

Program implementation and impact evaluation data were obtained from program reports or primary data records maintained by PELF.

## Results

### Disease burden and mapping

Early studies from the mid-20^th^ century summarized by Vincent *et al*. [15] documented MF prevalence ranging from 2%–7% in the capital Santo Domingo, with higher prevalence (8%–26%) found in rural areas surrounding the city. Vincent’s own work in 1980 found 8% MF prevalence from hospital in-patients in Barahona, in the southwest region, but zero infections out of 100 samples in the eastern town of La Romana. More extensive household studies from 1981–1985 recorded MF prevalence of 3.8% in Santo Domingo and surrounding areas, with prevalence significantly higher in males and adolescents 10–19 years old [10]. Geographically, infection was highest (9.8% MF positivity) in the urban *barrio* of La Ciénaga along the Ozama River. Vincent also reports contemporaneous unpublished surveys encompassing 3,566 samples from interior cities of Santiago, La Vega, Bani, San Juan and Pedernales that failed to detect any MF infections [10].

PELF began nationwide LF mapping in 1999 using a hybrid lot quality assurance sampling (LQAS) approach [16], in which a maximum of 250–300 children aged 6–10 years old from approximately five schools per municipality were tested for circulating filarial antigen (CFA) by immunochromatographic test (ICT; Binax, Inc.). If one positive sample was detected, the municipality was considered LF-endemic, and no further samples were collected. More than half (58%) of the country was mapped within the first two years with priority given to areas of suspected LF transmission based on historic data. Mapping was interrupted, however, in 2003 due to a global ICT shortage caused by a change in manufacturer and subsequent problems with test performance. Mapping resumed in early 2007 and was completed a few months later.

**Fig 1:**
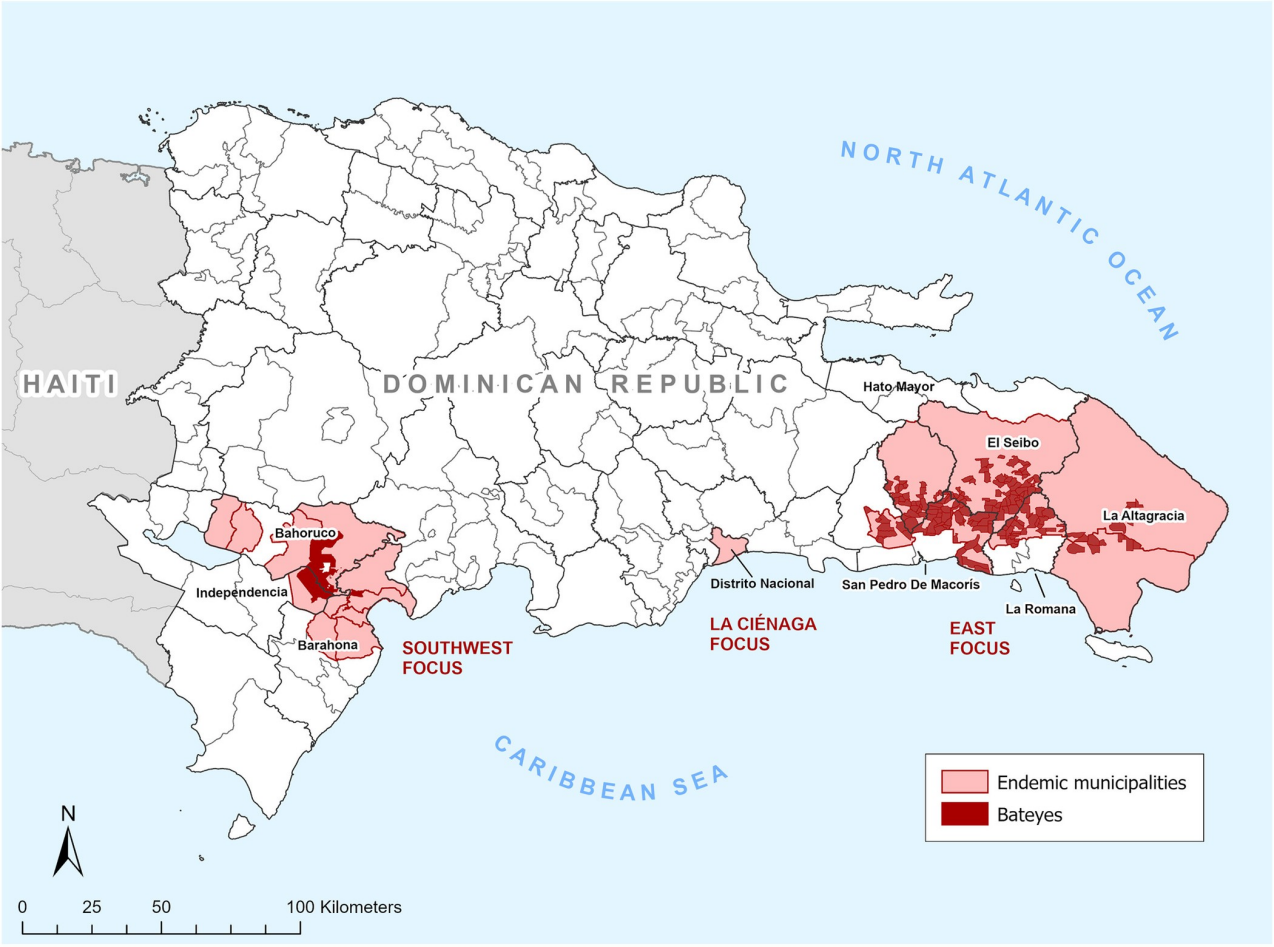
Map of the Dominican Republic showing LF-endemic areas (dark red) and associated municipalities (light red) identified from baseline mapping. The map was created using ArcGIS Pro version 2.8.1. No baselayer was used in the map. The source of the shapefiles is the Oficina Nacional de Estadística (ONE) of the Dominican Republic.

LF transmission was initially identified in 21 (14%) of the country’s 155 municipalities. However, two of these were considered non-endemic, as infections were detected only among non-permanent residents (temporary residents from other municipalities, or recent immigrants from Haiti). In the final analysis, 19 (12%) municipalities, with a total at-risk population of 262,395, were classified as LF–endemic and in need of MDA (Fig 1). The 19 municipalities were clustered in three geographic foci: the Southwest (10), La Ciénaga (1), and the East (8). The following sections detail the implementation of albendazole-DEC MDA to interrupt transmission and epidemiological monitoring that indicates WHO stop-MDA thresholds have been attained in each focus.

### Southwest focus

**Fig 2:**
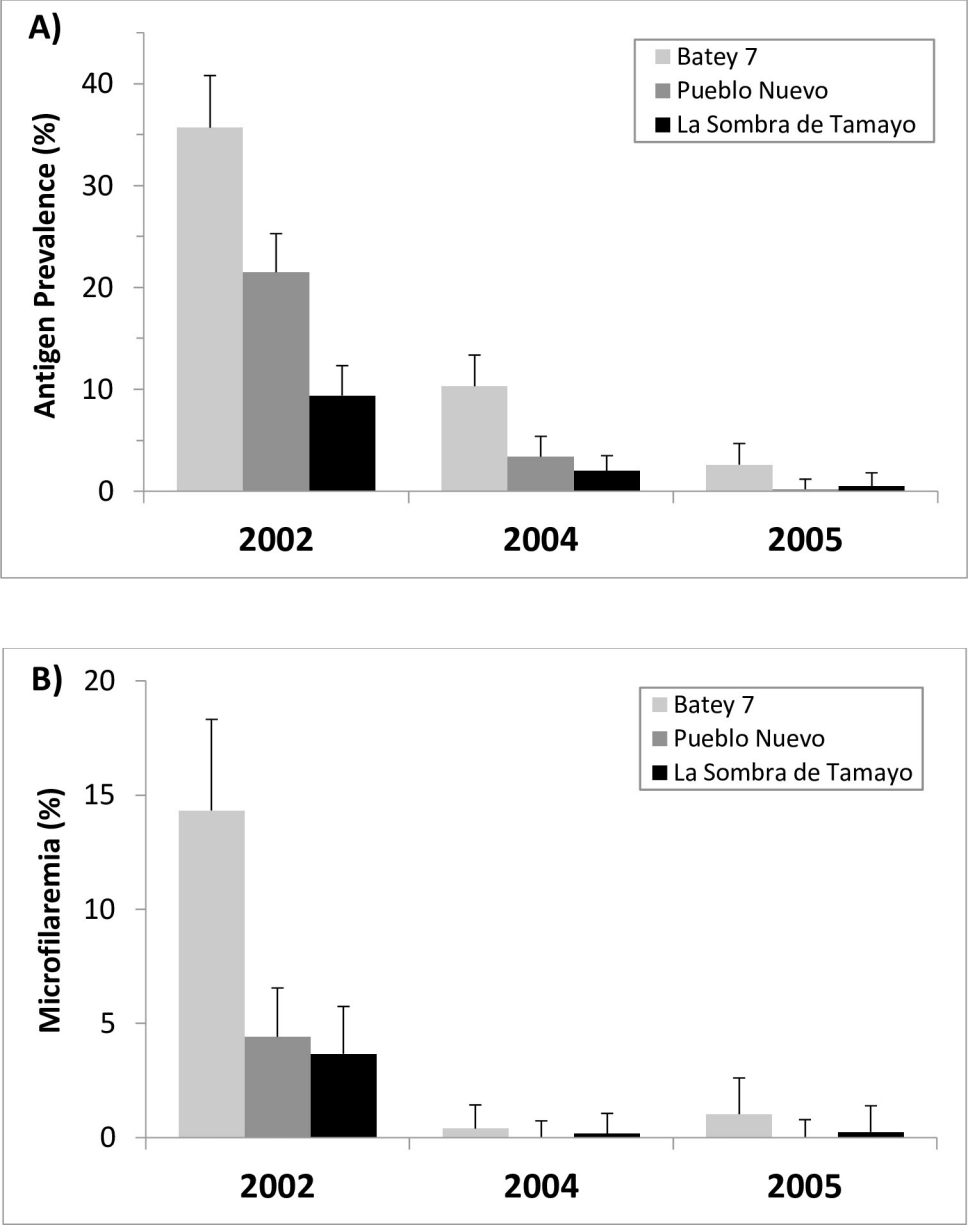
Prevalence, by year, of LF antigenemia by ICT test (A) and microfilaremia (B) in sentinel sites of the Southwest focus. Error bars show upper limit of 95% confidence interval.

The Southwest focus comprises 10 municipalities in three provinces (Independencia, Barahona, and Bahoruco) with an initial identified at-risk population of 145,957. The area forms a lowland valley surrounding the coastal city of Barahona that is irrigated by several inland lakes and rivers, and that historically supported a significant sugar cane industry. Before MDA implementation, PELF established sentinel sites in 2002 in each of the three provinces of the Southwest: Batey 7 in Independencia, Pueblo Nuevo in Barahona, and La Sombra de Tamayo in Bahoruco. Baseline CFA prevalence measured by ICT among individuals older than 5 years in these sites was 35.7%, 21.5%, and 9.4%, respectively, with MF prevalence of 14.3%, 4.4%, and 3.7%, respectively (Fig 2).

PELF spent a year training health workers for drug distribution, conducting a household census for MDA, and launching community mobilization. Drug distributors and supervisors were recruited from the local communities with a ratio of one distributor for every 50 target households and one supervisor for every four distributors. Distributors were required to be responsible, respected members of the community, while supervisors were local leaders who had participated in past community campaigns. Day-long trainings for distributors and supervisors were conducted in groups of 30 or fewer. All distributors and supervisors were required to take one dose of albendazole and DEC so that they would be sympathetic to complaints of side effects. Prior to MDA, PELF also conducted community awareness campaigns through school, church and other local meetings, house-to-house education, media messages using radio, television, and mobile loudspeakers in the targeted areas, and print media with photographs of lymphedema, hydrocele, and other clinical manifestations of LF. The purpose of these campaigns was to inform residents about LF, the drugs used for MDA, and the schedule of MDA campaigns in their community.

The first MDA in the Southwest took place in December 2002 (Table 1), with each of the 10 municipalities considered an implementation unit (IU). PELF worked with local leaders to select suitable dates and opted to conduct MDA over the weekend (Friday through Sunday) to maximize the likelihood of reaching people in or around their households. Drug distributors and supervisors traveled house-to-house to administer DEC and albendazole to eligible individuals (non-pregnant and older than two years of age). Extra health staff were stationed at local health centers during the weekend to attend to any adverse events. Drug distributors remained at health centers for two weeks after MDA to provide medication to those who were absent or initially refused (<1%). The first MDA in Southwest treated 117,544 persons—a reported coverage rate of 80.5% of the total population, while post-MDA coverage surveys found 86% of heads of households were treated. The estimated cost of the first MDA was USD $1.87 per person [17].

**Table 1:** Mass drug administration (MDA) for lymphatic filariasis in the Southwest focus.

MDA Round	1	2	3	4	5
**Year**	2002	2003	2004	2006	2007
**Dates**	Dec 13–15	Dec 13–14	Dec 10–11	Mar 20–Apr 1	Nov 19–30
**Implementation Units (municipalities)**	10	29	29	29	5*
**At-risk Population**	145,957	342,759	342,759	342,759	13,092
**Number Persons Treated**	117,544	250,759	237,215	271,815	11,873
**Epidemiological Coverage (%)**	80.5%	73.2%	69.2%	79.3%	90.7%

*only *bateyes*

Subsequent MDAs targeted an expanded population of 342,759 across 29 municipalities to treat communities adjacent to endemic municipalities. Despite more than doubling the target population, reported epidemiological coverage for the 2^nd^ (2003) and 3^rd^ (2004) MDAs was 73.2% and 69.2%, respectively (Table 1)—still above the 65% minimum coverage level recommended [8]. Coverage surveys in 2003 found 78% of households were treated. Baker and colleagues previously described how engagement with the local primary health care system—Unidades de Atención Primaria (UNAPs)—enabled this expanded effort [18]. Cost of the 2^nd^ MDA was estimated at USD $0.87 per person due to reduced start-up costs as well as devaluation of the Dominican Peso [17]. Funding limitations led to delays in the 4^th^ MDA, which did not take place until March 2006. Once conducted, the 4^th^ MDA included an extended 12-day house-to-house campaign in targeted areas, resulting in coverage of nearly 80% (Table 1). Sentinel site monitoring surveys conducted several months prior to the 3^rd^ and 4^th^ MDA campaigns, showed that CFA antigen prevalence among individuals older than five years of age declined by an average of 95% between 2002 and 2005 to ≤0.5% in Pueblo Nuevo and La Sombra de Tamayo and 2.6% in Batey 7, while MF prevalence also declined by 95% overall to 0% in Pueblo Nuevo in 2004 and 2005, 0.3% in La Sombra de Tamayo, and 1.0% in Batey 7 (Fig 2).

Criteria for stopping MDA proposed by WHO at that time included reduction of MF prevalence to less than 1% in adults [16]. Having attained this criterion in two of the three sentinel sites after three rounds of MDA, having conducted a high-coverage 4^th^ MDA in 2006, and faced with funding limitations and the disaster response to tropical storm Noel in 2007, PELF elected to limit the 5^th^ MDA to *bateyes*. Bateyes are settlement villages adjacent to agricultural plantations that historically house seasonal and long-term migrant workers, most of whom come from Haiti. This migratory pattern, coupled with the widespread distribution of LF in Haiti [11] and favorable ecological environment in *bateyes* for mosquitoes likely explains the concentration of LF transmission within these areas of the Southwest and Eastern foci in the Dominican Republic. The targeted *batey* population in the Southwest focus was 13,092, of whom 11,873 (90.7%) were treated in late 2007, the final year of MDA in the Southwest (Table 1).

WHO recommends a minimum four year period of post-treatment surveillance (PTS) following the cessation of MDA to confirm that LF prevalence remains significantly beneath sustainable transmission levels—believed to be 1% MF prevalence (2% antigen prevalence) in areas with *Culex* or *Anopheles* vectors [8]. At the time MDA was halted in the Southwest, global consensus had not been reached on the preferred methodology for PTS. PELF participated in a multi-country evaluation of the transmission assessment survey (TAS) by conducting community-based surveys in the 10 originally endemic municipalities in 2009 (two years after the last MDA) and in 2012 (five years after the last MDA) with the entire focus comprising a single evaluation unit (EU) [13]. TAS is a LQAS-type survey of children 6–7 years old, as this population was born during the MDA intervention period and should be free of infection if transmission has been interrupted. TAS-1 is conducted to make stop-MDA decisions, while repeated surveys (TAS-2 and TAS-3) are currently recommended for PTS. Since stop-MDA surveys were not conducted at the time of MDA halt in the Southwest, PELF conservatively considered the 2009 and 2012 surveys as TAS-1 and TAS-2, respectively. In 2009, none (0%) of the 1,692 children aged 6–7 years old tested by ICT in 38 randomly selected village clusters across the entire focus were CFA-positive, meaning that the Southwest passed TAS-1 (Table 2). PELF also simultaneously tested individuals older than 15 years in the same households. Out of 1,026 adults tested by ICT, only two CFA-positive individuals were identified (0.19% age group prevalence; 0.07% overall prevalence): one Dominican resident and a Haitian immigrant, both of whom were MF-positive. In 2012, five CFA-positive individuals (0.19% overall prevalence)—one adult (one of the ICT-positive individuals from 2009) and four children—were identified among 1,030 adults (0.10% age group prevalence) and 1,588 children (0.25% age group prevalence), respectively, tested by ICT in 40 randomly selected clusters. The antigen-positive adult and two of the four children were MF-negative. Three of the four CFA-positive children belonged to recently immigrated Haitian families, suggesting exposure outside the Southwest, while the fourth lived in a household that refused MDA. A result of four CFA-positive children was below the TAS critical cut-off of 18, meaning the Southwest focus also passed TAS-2.

**Table 2:** Results from transmission assessment surveys (TAS) in the Dominican Republic, by age and by focus area.

	*No*. *CFA-positive / No*. *Tested*, *% (95% CI)*					
	TAS-1		TAS-2		TAS-3	
	6–7 years	>15 years	6–7 years	>15 years	6–7 years	>15 years
**Southwest**	0 / 1692, 0% (0%–0.22%)	2 / 1026, 0.19% (0.02%–0.70%)	4 / 1588, 0.25% (0.07%–0.64%)	1 / 1030, 0.10% (0%–0.54%)	4 / 1620, 0.25% (0.07%–0.63%)	---
**La Ciénaga**	1 / 539, 0.19% (0%–1.03%)	---	0 / 815, 0% (0%–0.45%)	---	0 / 594, 0% (0%–0.62%)	---
**East**	1/ 1049, 0.10% (0%–0.53%)	---	---	---	---	---

In 2018, eleven years after the halt of MDA, community-based TAS-3 was again conducted in the 10 IUs of the Southwest. Of 1,620 children tested by filariasis test strip (FTS), four (0.25%) were CFA positive—significantly below the critical cut-off of 18 (Table 2). None of the antigen-positive individuals were MF-positive. However, in contrast to TAS-2 results, all four were resident Dominicans, raising the possibility of sustained low-level transmission in the Southwest. Follow-up investigations were not conducted surrounding the index cases.

### La Ciénaga focus

The second focus of LF transmission in the Dominican Republic is La Ciénaga. Meaning “swamp”, La Ciénaga is an impoverished urban area along the banks of the Ozama River in the capital Santo Domingo. Approximately 50,000 people live in La Ciénaga and the surrounding sub-*barrios* of Los Guandules and Guachupita. Surveys from the early 1980’s identified the area as a hot-spot of transmission with MF prevalence of 9.8% [10]. Pre-MDA sentinel site data from 2002 revealed CFA prevalence by ICT of 10.7% and MF prevalence of 2.5%.

The first MDA in La Ciénaga was conducted May to June 2004, with each sub-barrio considered an individual IU and an achieved coverage of 67.4% (Table 3). As in the Southwest, MDA was conducted over weekends and drug distributors and supervisors were recruited by PELF from within the community. However, unlike the largely rural Southwest, the urban environment of La Ciénaga presented different challenges: the density and geographical layout of households complicated census mapping and distribution, while reports of gang- and gun-related violence posed a perceived threat to the safety of program staff. To address these challenges, PELF partnered with a well-respected local non-governmental organization, Centro Juan Montalvo, to gain trust and instill community ownership. Details of this approach are reported elsewhere [19]. Additional keys to success were the organization and preparation of MDA staff. A total of 320 distributors and 64 supervisors were involved in each round of MDA distribution in La Ciénaga. They wore matching colored shirts and hats to increase visibility of the campaign. Once individuals were given medicine, a sticker was placed on the outside of their home, and the number of people ingesting treatment in that household was recorded. Stickers kept the PELF team organized in the midst of sprawling housing settlements, and also created a sense of ownership amongst the community members. Distributors were aware that non-residents would sometimes come from outside of the city to receive treatment during an MDA. To accurately enumerate the treated population and avoid inflation, distributors used different recording forms for residents and non-residents. Supervisors would individually meet with individuals who initially refused treatments to address any misconceptions about the drugs, provide additional education, and emphasize the importance of MDA in stopping transmission of LF within the community. MDA was repeated in 2005 and 2006, with reported epidemiological coverage of 92.2% and 85.8%, respectively (Table 2). Refusal rate was 1.3% or less in each round.

**Table 3:** Mass drug administration (MDA) for lymphatic filariasis in La Ciénaga focus, Dominican Republic.

MDA Round	1	2	3
**Year**	2004	2005	2006
**Dates**	May 21 –Jun 13	May 13 –Jun 05	Nov 17 –Dec 02
**Implementation Units (*sub-barrios*)**	3	3	3
**At-risk Population**	48,564	48,564	48,564
**Number Persons Treated**	32,715	44,761	41,660
**Epidemiological Coverage (%)**	67.4%	92.2%	85.8%

Sentinel site data in La Ciénaga collected prior to the 3^rd^ MDA in 2006 showed that antigen and MF prevalence among individuals greater than five years of age had been reduced to 0%. Therefore, PELF elected to stop MDA after three rounds based on these data and several other concomitant factors: 1) environmental improvements, initiated around 2003 in La Ciénaga, included street paving and covering of open sewers that reduced mosquito breeding sites; 2) albendazole monotherapy for treatment of soil-transmitted helminths (STHs) was given to school-aged children (5–14 years) starting in 2005, may also suppress *W*. *bancrofti* transmission [20].

Community-based surveys conducted in 2011, five years after the last MDA in La Ciénaga, found only one (0.19%) CFA-positive individual by ICT among 539 children aged 6–10 years tested in randomly selected households within the focus: a girl who lived in the area for less than two years (Table 2). Conservatively considering the 2011 survey as a stop-MDA survey, a school-based TAS-2 was conducted for PTS in 2014 with the entire focus considered a single EU. No CFA-positive samples were detected by ICT among 815 primary grade one and two children (approximating the 6–7 year age group) tested from each of the area’s seven schools in 2014 [14]. In 2018, a school-based TAS-3 was conducted using a similar study design. Zero (0%) of 594 children were CFA-positive by FTS, confirming transmission elimination in the area. The La Ciénaga experience provided empiric evidence that elimination of transmission can be achieved in areas of lower transmission with less than the 4–6 years of MDA currently recommended by WHO [8]. Fewer rounds of MDA resulted in program cost savings and prevented unnecessary drug administration to healthy individuals.

### East focus

The East focus is a low-land tropical area, with vast expanses of sugar cane and other agricultural industries that rely on migrant labor force as in the Southwest focus. Due to funding limitations and the lower intensity of transmission, the East was the last focus to initiate MDA. Sentinel site assessments conducted in 2011 revealed that transmission was limited to an at-risk population of 67,874 residing in *bateyes* of eight municipalities across five provinces (Hato Mayor, San Pedro de Macoris, El Seibo, La Romana, and La Altigracia). Mean CFA prevalence by ICT in sentinel sites (one per province) was 2.6% (range 0.6%–4.6%). MDA took place August to September 2014, when 52,854 people were treated resulting in epidemiological coverage of 77.9% (Table 4), despite funding shortages that limited pre-MDA social mobilization. Funding limitations also prevented the MDA planned for 2015. MDA resumed in 2016, this time accompanied by enhanced social mobilization that included community meetings, neighborhood announcements, and media distribution. A total of 55,879 people were treated in 2016 (85.1% coverage). A third treatment occurred in 2017, in which 56,985 persons were treated (81.7% coverage). Sentinel site monitoring conducted in 2018 with FTS revealed a 77% reduction in mean CFA prevalence to 0.6% (range 0.0%–1.3%).

**Table 4:** Mass drug administration (MDA) for lymphatic filariasis in the East focus, Dominican Republic.

MDA Round	1	2	3
**Year**	2014	2016	2017
**Dates**	Jul 18 –Aug 03	Sep 16 –Oct 02	Jul 07 –Aug 13
**Implementation Units (municipalities)**	8*	8*	8*
**At-risk Population**	67,874	65,674	69,718
**Number Persons Treated**	52,854	55,879	56,985
**Epidemiological Coverage (%)**	77.9%	85.1%	81.7%

*only *bateys*

Based on these results, the low baseline intensity of transmission in the East, and the absence of recrudescence in La Ciénaga after only three treatment rounds, PELF elected to conduct a stop-MDA TAS-1 in the East focus (considered as 1 EU) in 2018 after only three rounds DEC-albendazole MDA. Of 1049 children ages 6–7 years tested by FTS in community-based surveys, only one (0.1%) was CFA-positive: a 6-year old male, MF-negative, resident (Table 2). The area easily passed TAS against a critical value of 11. The halt of MDA in the East signaled the halt of MDA across all formerly LF-endemic areas of Dominican Republic.

### Post-treatment surveillance

Following the halt of MDA in the East region, a minimum of four years of PTS must occur for the country to fulfill WHO requirements for validation of elimination as a public health problem. The primary approach will be to conduct TAS-2 (scheduled for 2020 but delayed until 2021 due to COVID-19) and TAS-3 (scheduled for 2023) surveys in the East region. PELF also intends to conduct additional TAS in the other two transmission foci, even though they have successfully passed TAS-3. This is important given the presence of CFA-positive individuals detected in TAS-2 and TAS-3 surveys in the Southwest foci.

Another WHO-recommended approach for PTS is to incorporate LF testing with population-based surveys for other diseases [8]. In the Dominican Republic, a 2016 study evaluating risk of malaria and LF in *bateyes* across the Dominican Republic identified 6 CFA-positive individuals, none MF-positive, among 1418 individuals aged 2 years or older tested by FTS [21]. While three of the six were found in the Southwest (2) or East (1) endemic foci, the other three CFA-positive individuals were identified in ‘non-endemic’ areas: a Haitian migrant in Puerto Plata province in the north of the country and two individuals in adjacent survey clusters in San Cristobal province near the northwest border of Santo Domingo. These results highlight the importance of post-treatment surveillance throughout the country given population mobility across the border with Haiti and domestically (e.g. urban/peri-urban migration). For this reason, PELF intends to conduct a confirmatory remapping survey in historically ‘non-endemic’ areas following a similar approach from other countries [22]. Serological testing of samples from the 2016 *batey* survey is also underway to further inform LF transmission risk (Willingham et al., personal communication).

While LF-specific MDA has been halted across all endemic foci, other interventions likely maintain pressure against recrudescence. Albendazole monotherapy, which has modest effects in reducing MF levels [23], has been provided to school-aged children in the Dominican Republic for STH control annually since 2005 and semiannually since 2013. Additionally, nearly a quarter of a million long-lasting insecticide treated bed nets have been distributed in high-risk areas for malaria prevention since 2008. These exert mosquitocidal effects on susceptible *Anopheles* and *Culex* mosquitoes and provide personal barrier protection to block *Plasmodium* and *W*. *bancrofti* transmission.

### Morbidity management and disability prevention

Since its inception, PELF has aimed to alleviate suffering for LF patients, consistent with the GPELF twin pillar approach of transmission interruption through MDA and MMDP. Indeed, some of the earliest studies globally describing the psychosocial impact of LF were conducted in the Dominican Republic [3,24]. WHO recommends a ‘minimum package of care’ for LF patients that includes treatment for infection, access to hydrocele surgery, management of lymphedema, and treatment for episodes of acute adenolymphangitis (ADL) [25].

In 2001, a pilot hydrocele surgery program was established in which a cadre of three Dominican urologists (and two Haitian physicians) were trained in hydrocele surgery by Dr. Joaquim Norões. At least 52 hydrocelectomies were performed over the next two years. However, the project dissolved due to lack of sustained financial support. Morbidity surveys conducted in the Southwest (in 2002 and again in 2006) and in La Ciénaga (2004) identified 2,637 individuals with lymphedema and 256 cases of hydrocele, who were referred to their local health facilities or to the national Dominican Institute of Dermatology. While the latter provides specialized care to manage lymphedema and prevent ADL, WHO criteria for validation require that countries demonstrate the availability of care in each endemic area with known LF patients—along with estimating the number of LF patients in each IU and the readiness and quality of service in designated care facilities [5].

Therefore, the Dominican Ministry of Health needs to take several actions to meet WHO criteria: 1) Develop a national plan for LF MMDP. 2) Update estimates of the number of LF patients—particularly in the East region, where formal burden assessments have not been conducted. PELF plans to address this gap by including morbidity questions in PTS surveys and in the nationwide remapping survey planned for late 2020. 3) Establish designated care facilities in each area where LF patients are found.

## Discussion

In the two decades after its inception, the Dominican PELF distributed a total of 1,174,050 doses of DEC-albendazole in the country’s three endemic foci. By 2018, LF antigen prevalence was less than 2% in all foci, thus meeting criteria to stop MDA and begin post-treatment surveillance nationwide. This remarkable success has been achieved against a shifting landscape of limited domestic funding, competing public health priorities (including dengue, chikungunya, and zika virus outbreaks as well as frequent responses to tropical storms, hurricanes and other natural disasters), and sporadic external donor support.

Several key lessons emerge from PELF’s experience despite these limitations: 1) Baseline mapping data proved critical to prioritize areas for MDA, as scarcity of funds forced PELF to pursue a sequential approach to MDA scale-up. 2) Adaptability to local contexts was necessary as PELF encountered radically different environments between the rural *bateyes* of the Southwest and East foci and the urban setting of La Ciénaga. 3) Strong community engagement, taking different forms in each focus, was key to achieving high treatment coverage. In the rural foci of the Southwest and East, PELF’s outreach to, and work through, local community structures counteracted perceptions of exclusion and discrimination prevalent among area residents [26,27]. 4) Evidence-based decisions, forced by financial limitations, to stop MDA after fewer than five effective treatment rounds provided some of the first empiric data supporting the viability of this approach in low-transmission settings. 5) Continuity of leadership for the PELF program (since 2001) and a small, but dedicated team ensured unwavering commitment to program goals and approaches.

Remaining steps include the need to scale-up MMDP services for LF and to continue PTS. Assuming there remains no evidence of recrudescence or novel transmission, the Dominican Republic must continue PTS through at least 2022 (four years after the halt of MDA in the East region in 2018). However, as long as transmission occurs in neighboring Haiti, there is risk for imported infections, meaning that PTS should continue until transmission is interrupted island wide. During PTS, PELF intends to not only meet WHO criteria for elimination of LF as a public health problem, but also to collect evidence to support claims of verification of elimination of transmission, for which WHO is still developing criteria [28]. In so doing, PELF hopes to achieve its goal of LF elimination in the Dominican Republic, and to continue contributing to the global LF elimination learning agenda.
